# Factors Influencing Online Learning Satisfaction

**DOI:** 10.3389/fpsyg.2022.852360

**Published:** 2022-04-12

**Authors:** Qiangfu Yu

**Affiliations:** Faculty of Humanities and Foreign Languages, Xi’an University of Technology, Xi’an, China

**Keywords:** online learning satisfaction, online learner, online instructors, online platform, online instructional design, influencing factors

## Abstract

Online learning has received extensive attention in the field of education in the recent decade, especially after COVID-19 swept the globe in 2020. Online learning satisfaction (OLS) has become the focal point of the research, since it is of vital significance to enhance online learning efficiency. This paper reviews the research on OLS from the dimensions of online learners, online instructors, online platforms and online instructional design to have a clear picture of factors affecting OLS. Based on the review of previous studies, this mini review presents the prospect of future research on OLS and believes that breakthroughs on OLS research can be achieved by innovating research methods, expanding research subjects, and enriching research topics. OLS is a complicated dynamic system influenced by a diversity of factors, and it is worth more in-depth research by scholars and educators in future.

## Introduction

Online learning, a most significant aspect of education informatization development, has become the focus of attention in the field of education in the recent decade for its advantages of not being constrained by time, geographical location and other factors. In particular, COVID-19 swept the whole globe in 2020, posing unprecedented challenges politically, administratively, economically as well as pedagogically to the countries worldwide (Huang et al., 2020; Tan et al., 2021; Tlili et al., 2021). To effectively control and prevent the spreading of COVID-19, countries around the globe have been using online platforms to carry out online teaching and learning (Miller, 2020; Choi and Chung, 2021; Sobaih et al., 2021; Wlodarczyk et al., 2021). Policies like “suspending classes, ongoing learning” in China have been advanced and implemented, and online learning has been incorporated into every subject across almost all the school sectors globally, which has already made online learning an inevitable and irreversible trend in global education development (Cen et al., 2020). With this trend, an increasing diversity of online learning platforms have been adopted to facilitate online learning (Su and Chen, 2020). A large number of studies show that online learning can provide many positive learning experiences to online learners (Arbaugh, 2014; Eom et al., 2016; Li et al., 2016b,^2017^) and that learners are more satisfied with online learning than traditional face-to-face learning (Morton et al., 2016; Dooley et al., 2018; Green et al., 2018; Riddle and Gier, 2019). However, some studies show that online learning is not as satisfying as face-to-face learning and that online learners have poorer engagement with online learning (Pickering and Swinnerton, 2019). The possible factors accounting for poorer engagement and lower satisfaction are poor course design and poor pedagogy in online learning (Woodworth et al., 2015). The different arguments among scholars necessitates a more comprehensive, systematic and in-depth study of online learning satisfaction (OLS), which is of vital significance to enhance the service quality of online learning courses and perfect the online teaching quality evaluation system.

The term of online learning has been used in the field of education since the 1990s. It was first proposed to refer to placing some course materials on the computer networks to form a virtual learning community to achieve a face-to-face learning (Hiltz, 1999). Online learning is also known by some alternative terms like e-learning, blended learning, virtual learning, remote education, online education, web-based education, web-based instruction and online courses (Singh and Thurman, 2019), and there are some subtle differences in terminology not obvious for non-professionals to notice (Lee, 2017; Singh and Thurman, 2019). This article defines online learning to be anything from uploading learning materials onto some online learning platform to teaching and learning live through a diversity of software applications which facilitate “the bridging of the space between the teacher and the student through the use of web-based technologies” (Singh and Thurman, 2019, p.293).

Based on reviews of the literature on students’ satisfaction with online learning, this paper summarizes factors influencing OLS, and presents the prospect of future research on OLS.

## Online Learning Satisfaction

The concept of satisfaction has been long studied in the field of psychology (Myerson, 1943) and gradually expanded to other fields. Cardozo (1965) introduced customer satisfaction into the marketing field for the first time, which attracted great attention. Satisfaction was defined as the degree of pleasure felt by individuals, derived from their perceptions of product functions and their expectations for products (Kotler, 1997). Symonds (1955) explored what the field of education could learn from the field of psychology and learner satisfaction was mentioned in the discussion (Symonds, 1955). Since then, learning satisfaction has drawn great attention of researchers with different backgrounds, and different definitions of learning satisfaction have been provided. Learning satisfaction was defined as a feeling or attitude of learners that their desires and needs can be fulfilled in learning activities or processes (Houle, 1961; Long, 1989; Sanchez-Franco, 2009; Topala and Tomozii, 2014), a subjective psychological state formed after the comparison between the learner’s learning expectation and the actual perceived learning effect (Fernandes et al., 2013; Jiang et al., 2017a), and the learners’ evaluation of their satisfaction with teaching mode, course content, learning environment and other elements, as well as their learning state and learning effect (Xu, 2018). Based on the above definitions, this review paper holds the opinion that learning satisfaction is the feeling or attitude of learners toward learning activities, which directly reflects the degree to which learners’ expectations are fulfilled during the learning processes.

In order to monitor and improve the teaching and learning experiences, an increasingly large number of institutions and researchers have been employing student evaluation instruments to measure learners’ satisfaction levels (Arbaugh, 2014; Rienties, 2014; Asoodar et al., 2016; Bahati et al., 2019; Rajabalee and Santally, 2021). For example, the American Council on Education applied Cooperative Institutional Research Program in 1966 to measure the satisfaction of freshmen. Nowadays, a majority of institutions in the United States and the United Kingdom periodically collect learning satisfaction and academic performance data systematically (Baldwin and Blattner, 2003; Kember and Ginns, 2012; Rienties, 2014). And a number of scales targeted at learning satisfaction have been designed, among which the representative ones are the Students’ Evaluations of Educational Quality Questionnaire (Marsh, 1982), the Course Experience Questionnaire (Ramsden, 1991), the National Student Survey (Ashby et al., 2011; Callender et al., 2014), the Qualtrics Survey (Van Wart et al., 2020), and Performance Evaluation Matrix based on a fuzzy linguistic scale (Yu et al., 2018). These scales lay a foundation for the study on (online) learning satisfaction.

Online learning satisfaction refers to evaluation opinions and feeling experiences of learners toward the quality of online learning service provided by online learning providers, which is a cumulative psychological response to online learning contents and learning environment, formed after a rational and emotional comparison between the actual perceived online learning effect and expectations of the perception (Yao et al., 2016). Currently, OLS has become a focus of research drawing much attention (Bair and Bair, 2011; Ramayah and Lee, 2012; Ladyshewsky, 2013; Richardson et al., 2017; Alqurashi, 2019; Wang et al., 2019; Lin et al., 2020; Xiao and Li, 2021), especially after the spreading of COVID-19, among which the factors influencing OLS are the most hotly discussed (Hew et al., 2020; Zhang et al., 2020; Jiang et al., 2021; Zeng and Wang, 2021; Schroedler et al., 2022). Some of the factors of significance include the role of online educator (An et al., 2009; Costley and Lange, 2016), online interaction between teacher and learner (Baker, 2010; Kuo et al., 2014), perceived usefulness of online learning course (Liaw, 2008; Liu et al., 2015), online learning content (Kranzow, 2013), the role of platform technology (Dinh and Nguyen, 2020), learner’s motivation and efficacy (Artino, 2007; Alqurashi, 2019), online learning environment (Piccoli et al., 2001; Alqurashi, 2019) as well as assessment and evaluation systems (Dinh and Nguyen, 2020).

## Methods

In this mini review paper, previous studies on OLS were searched as follows. Firstly, studies published in international journals were searched in electronic databases of Web of Science, Elsevier, and Wiley Online Library. Take Web of Science for example. The author used the following searching parameters to conduct the search for previous studies: TI = (satisfaction) AND TI = (online learning OR online course OR online education OR remote education OR e-learning OR distance learning OR virtual learning OR distance education OR remote education OR blended learning OR web-based learning OR web-based education OR web-based instruction) AND SILOID = (WOS) AND PY = (2012–2022). Only studies exploring factors influencing OLS were extracted manually. Secondly, studies published in Chinese journals were searched in China National Knowledge Infrastructure (CNKI) database. The author used the Chinese counterparts of the searching parameters mentioned above and extracted the studies on OLS published in Chinese Social Sciences Citation Index (CSSCI) source journals.

## Results

As a result, 109 papers were found in Web of Science, 82 in Elsevier, 26 in Wiley Online Library, and 16 in CNKI. Among the 233 papers, 8 studies were published in 2012, 27 in 2013, 22 in 2014, 10 in 2015, 17 in 2016, 24 in 2017, 12 in 2018, 19 in 2019, 31 in 2020, 51 in 2021, and 12 in 2022. Based on a careful reading and analysis of the papers, this mini review summarizes the factors influencing OLS under four headings, namely, online learner factors (explored by 95 papers), online instructor factors (explored by 58 papers), online platform factors (explored by 74 papers), and online instructional design factors (explored by 85 papers).

### Online Learner Factors

Online learning satisfaction reflects the gap between the learners’ learning expectations and the actual perceived values. Individual characteristics of online learners, such as gender (Demei et al., 2013), age (Ke and Kwak, 2013), and self-efficacy (Kırmızı, 2015; Alqurashi, 2019; Han et al., 2021), will exert an impact on online learners’ previous expectations and perceived values, thus affecting their OLS.

Self-efficacy, a significant psychological construct in learning process (Alt, 2015), is defined as students’ beliefs in their capabilities to perform learning tasks (Alzubaidi et al., 2016). A number of studies have revealed a significant positive correlation between self-efficacy and OLS. Kırmızı (2015) found a positive correlation between students’ self-efficacy and OLS, supporting the findings of the study conducted by Chu and Chu (2010). Alqurashi (2019) investigated 167 online students and found that online learner self-efficacy was the strongest and most significant predictor of perceived learning, and a very important predictor of students’ OLS. What’s more, in a questionnaire survey about students’ stay-at-home online learning with a sample of 428 Chinese undergraduate EFL learners, Han et al. (2021) confirmed the significant mediating role of self-efficacy in students’ OLS.

What’s more, some other learner-related factors have been found in affecting OLS, such as student engagement (Gao et al., 2020; Han et al., 2021; She et al., 2021; Sinval et al., 2021), learner-learner interaction (Skinner et al., 2008; Kurucay and Inan, 2017), learner-instructor interaction (which is to be discussed in the following section), learner’s interaction with content (Knowles et al., 2020; Kim and Kim, 2021). What is noteworthy is the large scale study conducted by Li et al. (2016b). Using logistical regression modeling, the authors analyzed learning satisfaction data of 62,896 learners in 401 undergraduate online and blended modules and found that long-term goals of learners are important predictors of OLS (Li et al., 2016b). They also found that characteristics of individual learners only have a very minor impact on learning satisfaction, with one exception that older learners among new learners, especially those over 60, were 70% less likely to have high level of OLS, with the reasons to be further explored (Li et al., 2016b).

Whether some demographic variables of online learners like gender and age are closely related to OLS are controversial among researchers. Demei et al. (2013) surveyed 406 online students enrolled in an online course and also found that female students experienced higher level of OLS than male ones. However, through quantitative analysis of data collected from 392 students enrolled in 28 online courses, Ke and Kwak (2013) found that online learners’ age didn’t influence learners’ satisfaction. Hettiarachchi et al. (2021) also found no significant influence of gender and age on students’ OLS. Their finding may be affected by sampling biases in that the majority of the participants were female students, accounting for 88.3%, and male participants only accounted for about 11.7%. Another possible reason for the differences in their findings is differences in size of the samples, as Uttl et al. (2017), reanalyzing previous studies on the correlation between student evaluation of teaching (SET) ratings and student achievement, found that studies with large sample size showed no or only little correlation between SET ratings and student achievement, while studies with small sample size showed moderate and even large correlation.

### Online Instructor Factors

Whether in traditional face-to-face learning environment or in online learning environment, instructors play a most important role in the learning process of learners. Instructors’ attitude toward online learning, knowledge reserve, proficiency of teaching design, organization skills of teaching activities, and interaction with learners will greatly affect learners’ satisfaction with online learning. Costley and Lange (2016) adopted a quasi-experimental design to investigate the effects of instructor-control on learners’ OLS and found that instructor control of learning environments though instructional design could positively affect learners’ perceived OLS. Overall, instructors’ online teaching ability is the primary factor that affects learners’ OLS (Liu et al., 2015; Li et al., 2016a).

Interaction between learners and instructors is the most important factor determining OLS (Xu et al., 2017; Baber, 2020, 2021). Kuo et al. (2014) tested a regression model for OLS with 221 graduate and undergraduate students responding to an online survey. It was revealed that learner-instructor interaction and learner-content interaction were significantly predictive of student OLS but learner-learner interaction was not. And She et al. (2021) took a cross-sectional, questionnaire-based investigation of 1,504 Chinese university students and found a significant positive relationship between learner-instructor interaction and OLS. It was also revealed that learners’ self-efficacy and engagement serial mediated the relationship between learner-instructor interaction and OLS. What’s more, Yang (2014) studied the influence of teacher presentation ratio in video courses on learners’ OLS in his doctoral thesis. It was found that the proportion of teachers presented in video courses has a negative impact on learners’ satisfaction.

With the continuous development of big data technology and cloud computing technology, recent years have witnessed some larger-scale studies on OLS (Moskal et al., 2015; Uttl et al., 2017; Ullmann and Rienties, 2021). Langan and Harris (2019) analyzed over 1.8 million National Student Survey returns and found long-term stability of the predictors of OLS. It was found that the survey items related to “Teaching” was of significant influence, such as “Staff are good at explaining things,” “Staff have made the subject interesting,” and “Staff are enthusiastic about what they are teaching,” with the first item being particularly important (Langan and Harris, 2019), showing that the teacher and teaching factors are always more important than the learner factors. Besides, based on econometric modeling of 21,096 undergraduate student responses and 4,429 postgraduate student responses to the module evaluation questionnaire, Sutherland et al. (2019) found that the helpfulness of lectures and seminars provided by teachers, involving direct student-teacher contact time, is the most significant influencing factor of OLS. Similarly, in a large scale study of learning satisfaction of 16,670 new and 99,976 continuing students, Li et al. (2017) found that learners’ satisfaction with the assessment as well as advice and guidance provided by instructors were key factors influencing OLS of both new and continuing students.

### Online Platform Factors

Online learning can be classified into synchronous online course and asynchronous online course (Imsa-ard, 2020), the former being a real-time lecture provided through such video conferencing systems as ZOOM, Tecent Meeting and Webex Meetings and the latter being a pre-recorded lecture and some related course materials uploaded by the teacher onto such learning management systems as Blackboard and CANVAS (Oztok et al., 2013). OLS is formed after a comparison between the expectations and perceived cognition and emotions (Yao et al., 2016) generated in the interaction of leaners with those online learning platforms. Through a three-year study of 553 graduate and undergraduate students’ satisfaction with online learning, Cole et al. (2014) also found online learning platform, of which variables of importance included online distribution of learning materials, timely support services and user-friendly interface design, was a significant reason for satisfaction or dissatisfaction with online learning. Besides, Wang et al. (2014) conducted a questionnaire survey on 380 students and the results showed online course interface design had a significant impact on learners’ satisfaction and that the more user-friendly the platform interface design was, the more satisfied learners felt toward online learning.

There are many studies exploring technological means about online learning platform. Jiang et al. (2017b) made a comparative study of OLS in live situations and recording situations, and the results indicated that in live situations, learners tended to have a stronger sense of presence, but phenomena like video delaying and film not synchronizing with sound led to a lower level of OLS, while in recording situations, although video fluency was relatively higher, lack of interaction between instructors and learners made it difficult to establish emotional resonance, thus reducing OLS. And Zhang (2017) explored the influence of directory navigation on learners’ OLS and found that learners’ satisfaction was significantly higher in videos with directory navigation than in videos without directory navigation. Besides, Qian (2017) found that the average value of OLS of learners using barrage was relatively higher and sending barrage related to online learning contents could enhance students’ OLS.

### Online Instructional Design Factors

Besides student characteristics, instructor behaviors and learning platforms, instructional design is another significant determinant predicting OLS, which has been highlighted by a number of studies (Arbaugh, 2014; Sharples et al., 2014; Tobarra et al., 2014). Rienties et al. (2015), analyzing 40 learning designs at the Open University United Kingdom, found that the way online courses were designed had a significant impact on OLS, whereby learners’ satisfaction with online modules focusing on contents was significantly higher than online modules with a strong learner-centered focus.

What’s more, in a review of OLS of 62,896 learners, Li et al. (2016b) found that course design had a strong and significant influence on OLS for new learners and continuing learners. Learners who were more satisfied with the teaching materials, assessment strategies, and workload were reported to have a higher level of OLS (Li et al., 2016b). Similarly, in a review of over 1.8 million National Student Survey returns, Langan and Harris (2019) found that good organization and smooth running of the course was the most influential among all the factors influencing OLS. Besides, analyzing learning satisfaction of 116,646 students on 422 module designs with 232 variables across two academic years, Li et al. (2017) found learners’ satisfaction with online teaching materials was the most significant factor influencing OLS, observing that “the learners who were less happy with quality of teaching materials were 99% less likely to be satisfied (Li et al., 2017, p.12).”

## Discussion

Previous studies show that factors related to teachers and teaching are the most significant factors influencing OLS while factors related to learners are the least significant. However, previous empirical research on OLS collected data overwhelmingly through a combination of relatively smaller scale questionnaires and interviews, which may influence the validity of the research findings. To have a full and deeper understanding of OLS, an increasing number of large(r) scale studies are suggested to be conducted in future, with reference to some pioneering studies using large scale data (Li et al., 2016b,^2017^; Langan and Harris, 2019; Sutherland et al., 2019). It is also suggested that future studies might be integrated with techniques applied in cognitive neuropsychology, such as magnetoencephalography, electroencephalography and eye tracking technology, to obtain more scientific research conclusions.

In addition, most studies were carried out in a horizontal paradigm, that is, students’ OLS in a certain period was taken as the research object, and a scarcity of longitudinal studies were conducted on dynamic tracking investigation of OLS (Li et al., 2017). Since OLS is a dynamic value that changes over time and reflects the value of a continuous process (Li et al., 2017), longitudinal research from the developmental and ecological perspectives is suggested to be conducted on OLS to have a better and deeper knowledge of dynamicity of OLS and its relationship with a diversity of factors influencing OLS.

A large bulk of studies on OLS were focused on college students and college courses, with a few studies exploring OLS of primary school students and middle school students (Zhang et al., 2020). However, the booming development of online learning and the concept of lifelong learning attract people from all walks of life to participate in online courses, which makes online learning environment increasingly complicated. Different learners have different motivations for online learning, different expectation of support from online instructors, different perception of usefulness and convenience of online courses, and different levels of OLS. Therefore, the research subjects of future research should be expanded to focus on online learners from all social strata.

Research on OLS involves almost all aspects of online learning environment, but the previous studies were mostly focused on one or several factors that affect OLS (Baber, 2020), and ignored several factors like culture and context (Rubin and Fernandes, 2013). And since OLS is the result of the interaction of multiple factors, future research should be to explore the interrelationship of various factors, including culture and context, and the mechanism behind their interrelationship (She et al., 2021). Besides, previous research only focused on one type of online learning context, such as computers (Bhargava et al., 2021) and mobile phones (Xiao, 2021). Nevertheless, the fast development of technology has given creation to a diversity of digital media contexts (Kim et al., 2019). It is worth conducting research on OLS in different digital media contexts in future.

Finally, for the reason that the satisfaction degree is affected by a variety of factors, the results of OLS survey will inevitably show that some learners are satisfied while some are not, but there were few empirical studies concerned with intervention of OLS. Therefore, based on prior online satisfaction survey, future research should be conducted to find out the key factors leading to the variations in OLS of different learners, to develop online learning platform with adaptive technology and provide different learners with personalized and accurate online learning environments, and explore how learning design changes can enhance OLS of students (Li et al., 2016b).

## Conclusion

After elucidating some terms related to OLS, this mini review paper reviews and analyzes the research status of factors influencing OLS from the perspectives of learners, instructors, platforms and instructional design. It is suggested that large(r) scale studies be conducted to explore the diversity of factors influencing OLS of learners from all social strata and interrelationship of various factors, with techniques applied in cognitive neuropsychology. It is also suggested that longitudinal research be conducted on OLS to reveal the dynamicity of OLS, providing enlightenments on how to enhance online learning efficacy through intervention of OLS.

## Author Contributions

The author confirms being the sole contributor of this work and has approved it for publication.

## Conflict of Interest

The author declares that the research was conducted in the absence of any commercial or financial relationships that could be construed as a potential conflict of interest.

## Publisher’s Note

All claims expressed in this article are solely those of the authors and do not necessarily represent those of their affiliated organizations, or those of the publisher, the editors and the reviewers. Any product that may be evaluated in this article, or claim that may be made by its manufacturer, is not guaranteed or endorsed by the publisher.
